# Editorial: Editors’ showcase: Chemical biology

**DOI:** 10.3389/fchem.2022.1004363

**Published:** 2022-08-15

**Authors:** John D. Wade

**Affiliations:** ^1^ Florey Institute of Neuroscience and Mental Health, University of Melbourne, Melbourne, VIC, Australia; ^2^ School of Chemistry, University of Melbourne, Melbourne, VIC, Australia

**Keywords:** antimicrobial peptides, chemical biology, DnaK, exosomes, GPCRs, LC-MS, peptides, small molecule inhibitors

Today, chemical biology is a mature and widely recognized scientific discipline that seeks to translate knowledge of the structural and chemical basis of biology to better regulate or modulate biological processes. This can be achieved by a myriad of ways ranging from chemical synthesis, recombinant DNA production, biomolecular conjugation, selective detection, organic chemistry, structural and conformational analyses to affinity chromatography and proteomics This array of resources and tools available to the chemical biologist today can enable even the most complex of biomolecular interactions and pathways be studied and manipulated with incredible sensitivity and control.

In this special Research Topic, twelve leading international exponents of chemical biology together with their teams present their latest results across a wide spectrum of research. These highlight the importance of novel methods and technology together with the development of novel chemical compounds, be they small organic molecules, peptides, proteins, nucleic acids, lipids, glycans or hybrid molecules to better understand the chemical basis of biology and to use this knowledge for the preparation of more selective and potent mediators of biological function. The first of these by Matthyssen et al. examine the influence of multimerization (dimers, oligomer conjugates, dendrimers, polymers and self-assembly) of antimicrobial peptides (AMPs) on their potency and bacterial selectivity. Of particular importance is the role of such modifications against bacteria that are responsible for the production of antibiotic-resistant biofilms which remain the most difficult to prevent or treat.

AMP drug design and development is also the focus of the second contribution to this Research Topic. Here, Brakel et al. focus on the systematic structure-activity relationship study of the short proline-rich analogue ARV-1502 to determine the features that dictate binding to and inhibition of the bacterial chaperone DnaK. More than 180 synthetic analogues were prepared by chemical synthesis and assayed for activity against *E. coli* and *S. aureus*. Interestingly, those analogues that possessed increased inhibition of the DnaK chaperone system did not necessarily have increased antimicrobial activity which highlights the challenges associated with designing novel peptide-based antibiotics.

An alternative approach to the development of antibacterial agents is provided by Patil et al.. They describe the design and synthesis of AMP-peptide nucleic acid (PNA) conjugates as a means of targeting essential bacterial genes. Such antisense antimicrobials possess multimodal activity which is likely to confound the onset of antimicrobial resistance. Elegant organic chemical approaches to the conjugation for the chemically disparate AMP and PNA are investigated and used to develop conjugates that specifically target the acyl carrier protein gene of the Gram-negative bacteria *A. baumannii*. A cysteine-based click chemical method was shown to be most effective leading the way to the ready production of such compounds for antimicrobial drug development.

G protein coupled receptors (GPCRs), seven-transmembrane proteins, make up the largest family of cell surface receptors in mammalian cells and thus represent important drug targets with over 30% of FDA approved therapeutics. While there is good knowledge of the structural features that regulate GPCR action, comparatively much less is known about the role of post-translational modifications (PTMs) on receptor function. The manuscript by Zhang et al. describes the use of powerful mass spectrometry-based proteomics for profiling PTMs. The results shed important light on the role that PTMs play in increasing the functional diversity of GPCR regulation leading to new opportunities for selective GPCR-targeted drug development.

Xu et al. report in their manuscript the utility of modern and highly specific affinity capture strategies based upon molecular recognition for the isolation of exosomes, membrane extracellular vesicles that are secreted by all eukaryotic cells and which play critical roles in biological processes including cell-to-cell communication, immune response, and cell growth. The most common methods each have limitations which are described in detail, and which dictate careful consideration for use in specific applications. The ability to automate these methods currently remains beyond reach but is an attractive goal for the future.

The development of novel anticancer agents remains a subject of considerable research effort. Platinum-based drugs are of notable interest given their selectivity and site of action on DNA rather than proteins. Despite their effectiveness, non-selective cytotoxic effects and resistance development has motivated the development of new drugs. Rhenium based compounds were subsequently shown to possess potent anticancer activity with tricarbonyl rhenium isonitrile polypyridyl (TRIP) complex being notably effective. Yim and Park report the use of a label-free process, inductively coupled plasma mass spectrometry (ICP-MS), to conclusively demonstrate that TRIP acts on a protein, HSP60, and not DNA, to inhibit its chaperone function leading to cancer-specific cell death. The study highlights the importance of such tools for determining the molecular basis of metal-based drugs.

Post-translational modifications (PTMs) of peptides and proteins is a critical cellular process that adds significant structural and functional diversity. Glycosylation is arguably the most common PTM but is highly complex and generally leads to heterogenous glycan structures that are challenging to discriminate and characterize. The manuscript by Singh et al. reports the use of chemical synthesis of O-GalNac peptides to examine the influence of O-glycosylation on amyloid-β precursor protein (APP) proteolysis by β- and γ-secretases to produce amyloid-β peptides and possible subsequent Alzheimer’s disease pathology. The use of defined glycans obviates issues associated with typical glycosylation PTMs and enabled the authors to show that O-glycosylation can render APP model glycopeptides more susceptible to cleavage by β-secretase thus contributing to knowledge regarding the role of this PTM on amyloid-β aggregation.

The mammalian fibroblast growth factors (FGFs) are a family of 23 proteins that regulate a variety of cellular processes following interaction with one or more of 5 transmembrane receptor kinases (RTKs) known as fibroblast growth factor receptors (FGFRs). Abnormal FGF/FGFR interaction and signaling is frequently associated with tumor development including breast cancer and lung cancer. Consequently, the development of small molecule FGFR inhibitors has been an area of substantial research in the past 2 decades. The review by Zheng et al. provides and excellent survey of the early efforts to construct FGFR-targeting small molecules leading to subsequent *de novo* design of next-generation compounds with improved selectivity and potency. The roles of computer-aided design and, increasingly, artificial intelligence to expand the chemical space of potential inhibitors is discussed. Finally, the development and use of alternatives to small molecule inhibitors are described, notably PROTAC and molecular glues, and highlight the continuing importance of medicinal chemistry in oncology research.

One of the most important tools in chemical biology research is fluorescence microscopy in which a range of fluorophore labels are used to permit specific visualization of cellular structures and dynamics. Rozario et al. together with their colleagues report the development of improved single molecule (SM) super-resolution microscopy to undertake a remarkably detailed examination of the nuclear lamina of cultured COS-7 and T cells in 3D. The authors described the use of SM imaging using optical astigmatism together with multiplane acquisition and the photoswitchable fluorophore AlexFluor 647 to enable super-resolution discrimination of the entire nuclear lamina morphology leading to quantification of overall nuclear dimensions and local membrane features.

Greule et al. describe the use of combination of approaches including chemical synthesis, structural biology, biochemistry, enzymatic catalysis and protein engineering to examine the structure and function of the monooxygenase, cytochrome P450 OxyA from kistamycin. The enzyme plays a significant role in the cellular glycopeptide antibiotic biosynthesis. It was revealed that the precise heme orientation is crucial to enzyme function which, in turn, highlights the great potential of P450s for use as biocatalysts to perform synthetically challenging transformations both *in vitro* and *in vivo* and to produce new medically important bioactive compounds.

Demyelination is a hallmark of multiple sclerosis (MS) although numerous debates continue as to its cause. It is generally agreed that a T-cell mediated inflammatory process directed against myelin is a critical contributory event. Myelin basic protein (MBP) is a major component of the myelin sheath and is thought to be a target by antibodies which may lead to the pathogenicity observed in MS. To contribute to a better understanding of the possible role of MBP in the immune response, Staśkiewicz et al. undertook to chemically assemble several peptide fragments of MBP and to measure their association with IgM antibodies derived from MS patient sera and to correlate the findings with the helical conformation of the peptides. They showed that such synthetic antigenic probes can be valuable tools for discriminating between different ELISAs and subsequent further understanding of the role of MBP in MS.

The role of peptide hormones in health and disease requires sophisticated and sensitive detection and quantification methods which has typically been afforded by immunoassay. The necessity for increased sensitivity has led to the development of mass spectrometry methods although the reproducibility and reliability of these have yet to enable their ready utility. The manuscript by Hering et al. report a systematic evaluation of sample preparation as a critical component of peptide quantification using tandem triple quadrupole mass spectrometer together with peptide modification with a quaternary pyridinium ion. Such approaches enabled a substantial improvement in quantifying serum oxytocin levels which in turn will aid research into the role of this hormone in psychiatric disease.

Together this collection of contributions highlights the continuing and powerful role chemical biology makes in our understanding of complex cellular processes and in the development of novel tools, diagnostics and therapeutics. It is hoped that the reader will enjoy and appreciate this Research Topic.

